# Application of a Homogenous Assay for the Detection of 2,4,6-Trinitrotoluene to Environmental Water Samples

**DOI:** 10.1100/tsw.2005.58

**Published:** 2005-05-23

**Authors:** Ellen R. Goldman, Adrienne L. Egge, Igor L. Medintz, Michael E. Lassman, George P. Anderson

**Affiliations:** U.S. Naval Research Laboratory, Center for Bio/Molecular Science and Engineering, Washington, DC 20375, USA

**Keywords:** homogeneous assay, 2, environmental monitoring, explosives, fluorescent displacement immunoassay

## Abstract

A homogeneous assay was used to detect 2,4,6-trinitrotoluene (TNT) spiked into environmental water samples. This assay is based on changes in fluorescence emission intensity when TNT competitively displaces a fluorescently labeled, TNT analog bound to an anti-TNT antibody. The effectiveness of the assay was highly dependent on the source of the sample being tested. As no correlation between pH and assay performance was observed, ionic strength was assumed to be the reason for variation in assay results. Addition of 10x phosphate-buffered saline to samples to increase their ionic strength to that of our standard laboratory buffer (about 0.17 *M*) significantly improved the range over which the assay functioned in several river water samples.

## INTRODUCTION

Munitions storage and processing has led to the contamination of soil and groundwater with the explosives, including 2,4,6-trinitrotoluene (TNT)[1,2]. Cleanup of contaminated areas has generated a need for rapid, selective, and reliable methods for on-site quantification of explosives[3]. Areas in soil, groundwater, and seawater contaminated with TNT from past manufacture, storage, and demilitarization of weapons are monitored to identify the extent of the contamination. On-site analysis methods can be used both to assess the nature and extent of contamination, and to monitor remediation progress.

Many of the currently available on-site methods to monitor TNT involve multiple steps and require between 20 min and 2 h[3,4,5,6,7,8,9]. Other methods provide results in only a few minutes, but are not designed for high sample throughput[10].

We had previously developed a homogeneous assay for the detection of TNT based on the fluorescence intensity change of a dye-labeled TNT analog in its antibody bound and free states[11,12]. An increase in fluorescence occurs when anti-TNT antibody binds the fluorescent TNT analog. TNT concentrations in aqueous samples are determined by monitoring the fluorescence decrease as unlabeled

TNT in the test samples competes with the fluorescent TNT analog for binding by the antibody. This assay has the advantages of being fast, simple, and sensitive, all qualities essential for adaptation to a high-throughput format. In the current work, we demonstrate that this assay format works with an alternative dye-labeled analog and apply it to TNT spiked into environmental water samples.

## EXPERIMENTAL

### Materials

Alexa Fluor® 555 cadaverine, disodium salt was purchased from Molecular Probes (Eugene, OR). The anti-TNT antibody 30-1[13] was used for all tests described. TNT standards were from Cerilliant corporation (Round Rock, TX). Artificial seawater was prepared by mixing 40 g seasalts (Sigma, St. Louis, MO) per 1 l of deionized water. Environmental water samples were collected from the designated sites into clean, sterile tubes and kept refrigerated.

### Construction of TNB-Alexa Fluor 555

One milligram of Alexa Fluor 555 cadaverine, disodium salt was dissolved in 500 μl of borate buffer (0.136 *M* Na tetraborate buffer pH 8.5) and 350 μl of 5% 2,4,6-trinitrobenzenesulfonic acid solution (Sigma) was added with 20 μl of 3 *M* NaOH and the solution was reacted overnight at room temperature with agitation and then loaded on a Supelclean™ LC-18 SPE column (Supelco, Bellefonte, PA), washed with 0.1× borate buffer, and eluted with an increasing concentration of methanol in borate buffer. Eluted fractions were desalted and purified on an oligonucleotide purification cartridge (OPC, Applied Biosystems, Foster City, CA) as described[14] and the synthesis of the final Alexa Fluor 555-trinitrobenzene (AF-TNB) product was confirmed with mass spectral analysis.

### Assay

The assay was carried out essentially as described previously[12]. Briefly, white 96-well plates (Maxisorp, Nunc) were blocked overnight with 300 μl of a blocking solution consisting of 1% bovine serum albumin (BSA) in Tris-buffered saline (TBS)(50 m*M* Tris-HCl, 105 m*M* NaCl, pH 7.5) at 4$^{o}$C. The blocking buffer was discarded and 25 μl of 100 n*M* AF-TNB analog plus 25 μl of 5 μg/ml 30-1 anti-TNT antibody[13] was added to each well. TNT dilutions between 0.1 and 10,000 ng/ml TNT standards into PBS, artificial seawater, or environmental water solutions were prepared. A 50-μl aliquot of each standard TNT dilution, or no TNT as a control, was then added to appropriate wells. Sample fluorescence was read within 5 min after final plate preparation. Samples and controls were performed in at least triplicate. Fluorescence emission was read using a Tecan Safire plate reader (Tecan US, Research Triangle Park, NC) with 555-nm excitation and 575-nm emission. A 5-nm bandwidth was chosen for both the emission and excitation wavelengths. The data were plotted as the difference of the average signal in the control wells that contained no TNT and the average fluorescent signal in the sample wells.

## RESULTS AND DISCUSSION

### Use of the AF-TNB

Previously, we had used a Cy5-DAP-TNB, TNT analog, in the homogeneous assay format[11]. As part of the current work, we investigated the use of another dye-TNB reagent. We found that the AF-TNB

provided a more simple synthesis than the Cy5-DAP-TNB, as the Alexa Fluor dye was purchased with an amine linker. In addition, substituting the AF-TNB analog in the assay provided identical limits of detection as with the Cy5-based analog. As had been seen with the Cy5-DAP-TNB[11], the homogeneous assay using the AF-TNB performed as well in artificial seawater as in phosphate-buffered saline (PBS) (Fig. 1).

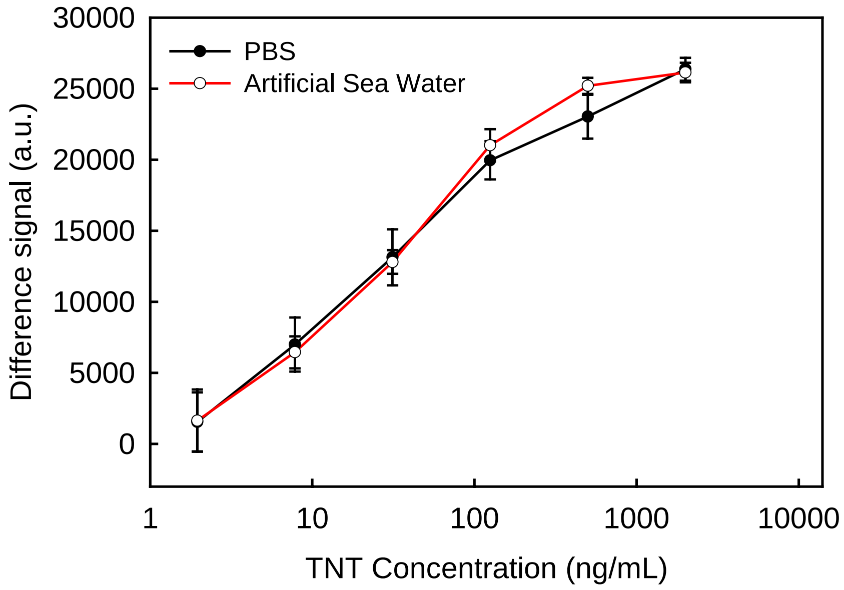

**FIGURE 1**. Results from TNT standards spiked into PBS and artificial seawater. Each point is the average of three measurements and the error bars represent the standard deviation.

## Environmental Water Samples

We examined samples from environmental waters including lakes, rivers, and the ocean to assess how the homogeneous assay functioned in these matrices. When testing nonbuffered solutions, all solutions (the AF-TNB, 30-1 antibody mix, and the TNT samples) were made up in the environmental water sample.

Performance varied dramatically depending on the water sample in which the assay was run. Fig. 2 shows the results of TNT standards spiked into PBS, water from the coast of Bahrain, Pusan Harbor, and Lake Michigan. The best signal was found in the ocean water from Bahrain, followed by PBS, Pusan harbor, and finally, Lake Michigan, which only showed signal at the highest TNT concentration. Fig. 3 shows signal from TNT spiked into river water samples. In this data set, TNT spiked into the Connecticut River gave almost as strong a signal as TNT spiked into PBS. This water sample was taken close to the mouth of the river where it runs into the Long Island Sound. No signal was seen in TNT spiked into the Rhappahannock River water, and signal was only seen at the highest concentration when water from the Shenandoah River was tested. The Potomac River water showed a signal profile in which little change was seen with increasing TNT concentrations until the highest concentration of spiked TNT.

No correlation was found between the pH of the water samples and assay performance. Water samples showed values of pH between 6.8 and 10. Examination of the data seemed to indicate that the assay performed best in water with presumed higher ionic strength. The two river waters that had performed the poorest in the assays were retested with the addition of 10× PBS to bring the final ionic strength of the sample to at least that of our laboratory PBS buffer (about 0.17 *M*). Fig. 4 shows that assay performance was improved in both river waters. The Rappahannock water performed the same as PBS on the addition of buffer. The performance of the assay in the water from the Shenandoah River was also improved with the addition of buffer.

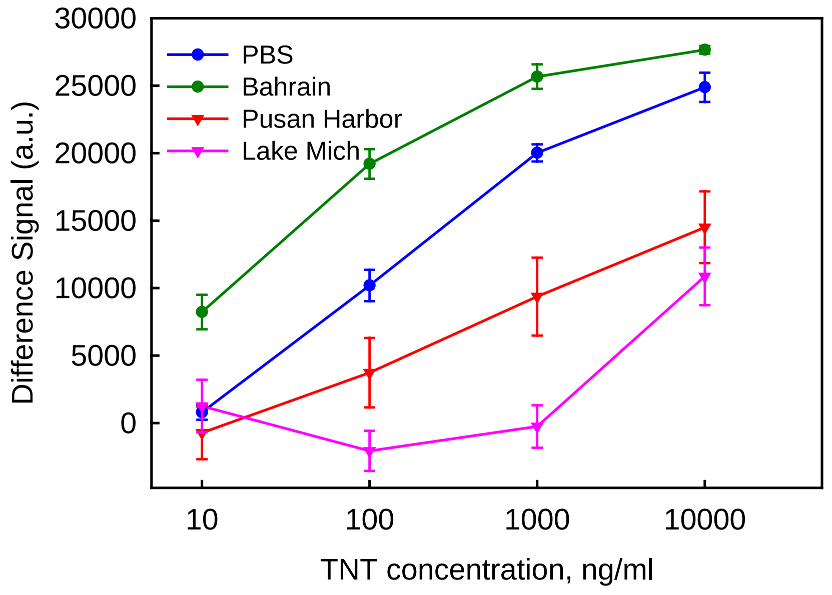

**FIGURE 2**. Results from TNT spiked into assorted environmental water samples as well as PBS. Each point is the average of three measurements and the error bars represent the standard deviation.

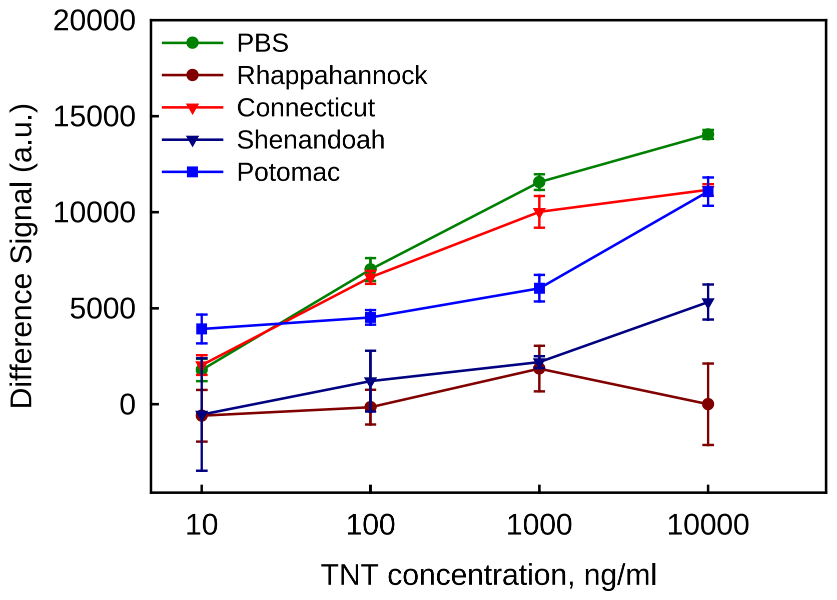

**FIGURE 3**. Results from TNT spiked into various river waters as well as PBS. Each point is the average of three measurements and the error bars represent the standard deviation.

Other properties of the tested water may also contribute to assay performance. For example, it has been found that high amounts of organic materials in water may compete with TNT for the antibodies binding site[15]. Antibody-based methods for TNT detection have previously been shown to exhibit matrix effects when complex environmental samples with multiple contaminants were tested[16]. It is important to consider that in addition to the ionic strength of the water being tested, other components of the matrix, such as high humic content, may interfere with the assay.

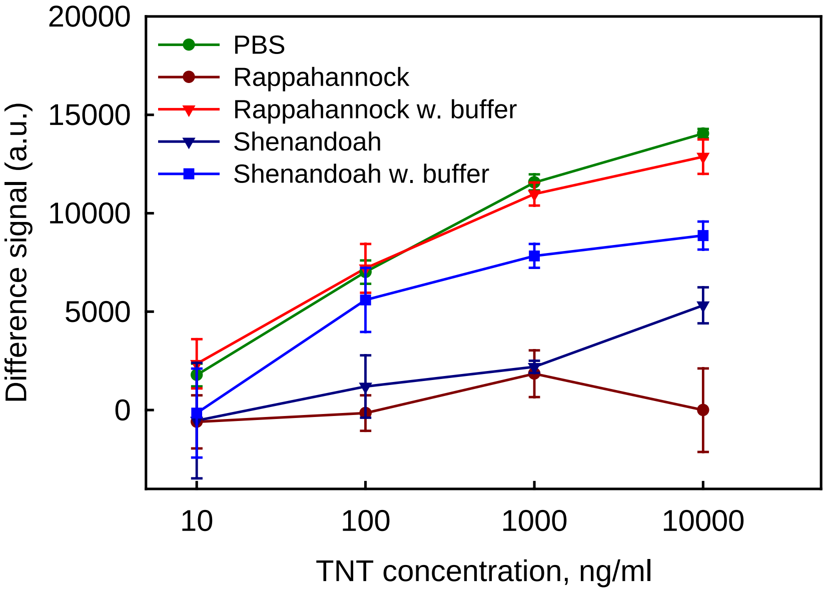

**FIGURE 4**. Results from TNT spiked into river waters with and without the addition of 10× PBS to bring the overall ionic strength of the solution to that of PBS. TNT spiked into PBS is also shown on this plot. Each point is the average of three measurements and the error bars represent the standard deviation.

## CONCLUSIONS

We have demonstrated that our homogeneous assay for the determination of TNT can be successfully applied to environmental water samples and performs particularly well in salt water. The assay behaves differently, however, depending on the characteristic of the sample. It is therefore imperative to run TNT standards to construct a standard curve in the solution in which the measurements are to be made. As mentioned, the assay seems to function better in samples with a higher ionic strength. Addition of 10× PBS to bring the ionic strength up to that of the laboratory PBS buffer seems to improve the range over which the assay functions in several samples of river water.

## ACKNOWLEDGMENTS

MEL is supported by an American Society for Engineering Education (ASEE) fellowship. ALE was mentored through the science and engineering apprenticeship program supported by a DOD grant. This work was funded by the Office of Naval Research. The views, opinions, and/or findings described in this report are those of the authors and should not be construed as official Department of the Navy positions, policies, or decisions.
